# Sub-Inhibitory Cefsulodin Sensitization of *E. coli* to β-lactams Is Mediated by PBP1b Inhibition

**DOI:** 10.1371/journal.pone.0048598

**Published:** 2012-11-06

**Authors:** Sujoy K. Sarkar, Mouparna Dutta, Akash Kumar, Dhriti Mallik, Anindya S Ghosh

**Affiliations:** Department of Biotechnology, Indian Institute of Technology, Kharagpur, West Bengal, India; The Scripps Research Institute, United States of America

## Abstract

The combination of antibiotics is one of the strategies to combat drug-resistant bacteria, though only a handful of such combinations are in use, such as the β-lactam combinations. In the present study, the efficacy of a specific sub-inhibitory concentration of cefsulodin with other β-lactams was evaluated against a range of Gram-negative clinical isolates. This approach increased the sensitivity of the isolates, regardless of the β-lactamase production. The preferred target and mechanism of action of cefsulodin were identified in laboratory strains of *Escherichia coli*, by examining the effects of deleting the penicillin-binding protein (PBP) 1a and 1b encoding genes individually. Deletion of PBP1b was involved in sensitizing the bacteria to β-lactam agents, irrespective of its O-antigen status. Moreover, the use of a sub-inhibitory concentration of cefsulodin in combination with a β-lactam exerted an effect similar to that one obtained for PBP1b gene deletion. We conclude that the identified β-lactam/cefsulodin combination works by inhibiting PBP1b (at least partially) despite the involvement of β-lactamases, and therefore could be extended to a broad range of Gram-negative pathogens.

## Introduction

The use of antibiotics in optimum concentration is among the most desirable criteria for any treatment strategy [1]. Reports suggest that the usage of cefsulodin, a third-generation cephalosporin, in combination with other β-lactam antibiotics, such as cefazolin and cefuroxime, produces a synergistic effect on sensitivity alteration [2]. However, the optimal concentration of cefsulodin is not known and therefore, narrowing down the concentration will facilitate treatment with comparatively lower doses of antibiotics.

Cefsulodin is thought to target penicillin-binding proteins (PBPs) 1a (encoded by *mrcA*) and 1b (encoded by *mrcB*), which are bifunctional enzymes with transglycosylase and transpeptidase activity [3]; but, it is not known which one of the two PBPs is the preferred target. Reports [4], [5] claim that PBP1b deletion alters β-lactam sensitivity; however, the reversal of sensitivity by PBP1b complementation has not been established. In addition, much of the work in this area has been conducted on *E. coli* K-12 laboratory strains devoid of O-antigens. As O-antigen plays a significant role in β-lactam sensitization [6], it is important to study its effect in the context of PBP1b deletion.

Some β-lactams that target a specific PBP produce an effect similar to the deletion of that PBP, for e.g. inactivation of PBP1 by cefsulodin [5], [7]. Here, we report a combination of cefsulodin with other β-lactams that acts synergistically in sensitizing laboratory and clinical isolates, regardless of the presence of β-lactamases and/or O-antigens.

## Materials and Methods

The clinical isolates used for the study were collected from the Tropical School of Medicine, Kolkata, India. These isolates were identified using a combination of biochemical tests and 16S ribosomal DNA sequencing. The obtained sequences were matched with the sequences in the database (National Center for Biotechnology Information), and sequences with a more than 98% match were taken into consideration (as there were no new sequence results, they have not been deposited into Genebank). Primarily, the strains were screened for the presence of β-lactamase by assessing the ability of the cell lysates to hydrolyze nitrocefin [8], and thereafter, specifically for CTX-M β-lactamases (most widespread extended-spectrum β-lactamase) by using polymerase chain reaction with specific primer pairs [9]. The minimum inhibitory concentration (MIC) values of the antibiotics from both penicillin and cephalosporin groups were determined according to CLSI guidelines either individually or in combination [6], [7], [10]. Bacterial strains used for genetic manipulations were derived from the *E. coli* K-12 strains, CS109 (O-antigen negative strain) and 2443 (O-antigen positive strain). The PBP genes *mrcA* and *mrcB* were deleted by P1 transductions followed by the excision of the *res–npt–res* cassette by transient expression of the RP4 ParA resolvase [7], [11], [12]. Strains and plasmids used in this study are listed in Table 1.

**Table 1:** *Escherichia coli* strains and plasmids.

Strain/plasmid	Genotype/relevant features	Source
CS109	W1485 *rpoS rph*	C. Schnaitman
AM1A-1	CS109Δ*mrcA*	This work
AM1B-1	CS109Δ*mrcB*	This work
2443	*thr-1 leuB6* Δ(*gpt-proA*)*66 argE3 thi-1 rfb_O8_ lacY1 ara-14 galK2 xyl-5 mtl-1 mgl51 rpsL31 kdgK51 supE44*	A.T. Maurelli
AM1OA-1	2443 Δ*mrcA*	This work
AG1OB-3	2443 Δ*mrcB*	This work
pJMSB8	RP4::2-Tc::Mu-Km::Tn7] λ pir lysogen (Amp^R^)	[12]
pSAD588-1	*mrcB* cloned in pBAD18-Cam	K.D. Young
pFS1A1	*mrcA* cloned in pBAD18-Cam	K.D. Young

PBP deletion was confirmed by labeling the cells with Bocillin FL (Invitrogen Inc., Carlsbad, CA, USA) and visualizing the PBPs separated in SDS-PAGE [13]. The PBP1a and 1b clones (pFS1A1 and pSAD588-1, respectively) were gifted by Professor Kevin D. Young. MIC values were determined for *E. coli* CS109, 2443 and their deletion mutants, before and after complementation. To check the β-lactam binding efficacy of PBP1a and 1b, competition assays between cefsulodin and Bocillin FL were performed, using *mrcA* and *mrcB* deleted strains [8]. The killing kinetics was evaluated following the methods described earlier [14].

**Figure 1:**
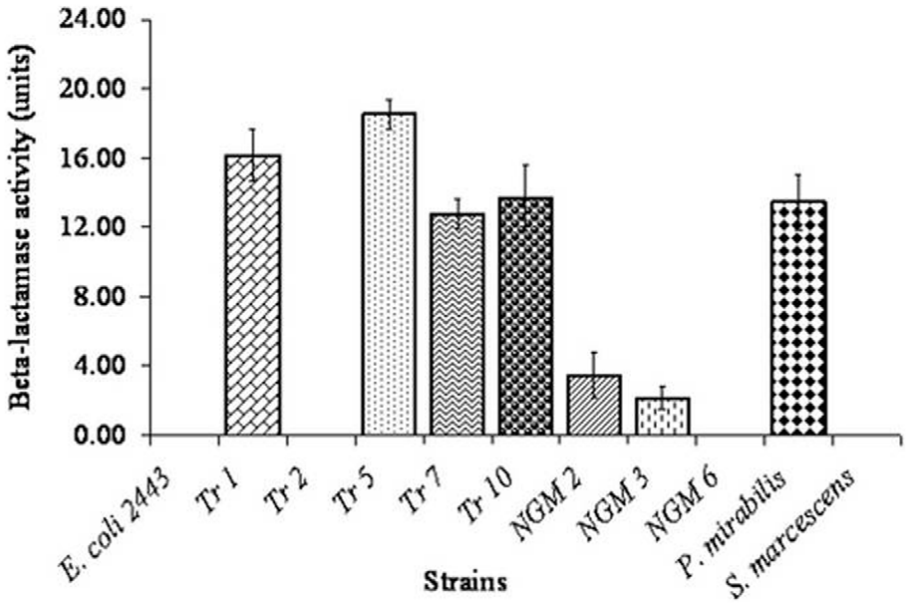
β-lactamase assay for various strains used in this study. β-lactamase activity could not be detected for Tr2, NGM6 and *Serratia marcescens*.

## Results and Discussion

### Sub-inhibitory Concentration of Cefsulodin Combination Sensitizes Gram-negative Clinical Isolates

Clinical isolates selected for this study were identified as the members of *Enterobacteriaceae* group. The strains Tr1 (*Salmonella enterica*), Tr5 (*Shigella* sp.), Tr10 (*Klebsiella pneumonia*), Tr7 (*Escherichia coli*), NGM2 (*Escherichia coli*), NGM3 (*Escherichia coli*) and the type strain NCIM 2300 (*Proteus mirabilis*) showed significant β-lactamase activity while the strains Tr2 (*Vibrio cholera*), NGM6 (*Escherichia coli*), and the type strain NCIM 2397 (*Serratia marcescens*) lacked β-lactamase activity (Figure 1). Susceptibilities of these strains to various β-lactams were tested and the MICs varied from 2 mg/L to >500 mg/L (Table 2). When combined with cefsulodin, at concentrations ranging from 2 mg/L to 8 mg/L, susceptibilities of all the strains were enhanced. However, differences in susceptibilities were negligible when cefsulodin was used at concentrations higher than 4 mg/L. The combination successfully sensitized the strains 2 to 32 fold compared to their original MIC values (Table 2). Therefore, the combination of cefsulodin at a sub-inhibitory level (4 mg/L) with other β-lactam agents was effective against both β-lactamase negative and positive strains. For further experiments, the concentration of cefsulodin used in the combination was 4 mg/L.

**Table 2:** β-lactam^a^ sensitivities of bacterial isolates in combination with Cefsulodin (4 mg/L).

Strains	AMX	AMX+CSN	PIP	PIP+CSN	AMP	AMP+CSN	PNG	PNG+CSN	CDL	CDL+CSN	CLN	CLN+CSN	CXN	CXN+CSN	CCR	CCR+CSN	CZM	CZM+CSN	CTX	CTX+CSN	CSN
*Proteus mirabilis* NCIM 2300	2	0.5	4	0.5	2	0.5	16	2	8	1	8	0.5	2	0.25	2	0.25	2	0.25	1	0.06	16
*Serratia marcescens* NCIM 2397	16	4	8	1	16	4	125	32	4	0.5	16	0.5	4	1	4	1	2	0.5	2	0.5	32
*Vibrio cholerae* Tr2	16	4	0.25	0.03	16	4	64	16	16	4	16	4	4	1	2	0.5	1	0.25	2	0.25	32
*Shigella sp.* Tr5	125	64	8	2	125	64	>500	>500	16	4	16	2	8	2	4	1	0.5	0.06	0.5	0.03	32
*Klebsiella pneumoniae* Tr10	500	125	16	2	250	64	500	250	16	4	16	2	8	2	2	0.5	1	0.125	0.5	0.03	32
*Salmonella typhimurium* Tr1	250	125	16	4	250	125	250	125	16	4	16	4	8	2	4	1	1	0.125	0.5	0.06	64
*Escherichia coli* Tr7	250	125	8	2	250	125	500	250	16	4	32	16	4	1	4	1	4	0.25	0.5	0.06	32
*Escherichia coli* NGM2	32	16	ND	ND	ND	ND	125	64	ND	ND	32	8	16	4	4	1	4	0.25	1	0.125	32
*Escherichia coli* NGM3	16	8	16	1	8	2	250	125	125	16	16	4	16	4	4	1	4	0.5	2	0.25	32
*Escherichia coli* NGM6	16	8	16	2	4	2	250	125	32	8	32	16	4	1	4	1	4	0.5	1	0.125	32

a AMX  =  amoxicillin; AMP =  ampicillin; PIP  =  piperacillin; PNG  =  penicillin G; CDL =  cefadroxil; CLN =  cefalexin; CCR =  cefachlor; CXN =  cefoxitin; CML =  cefamandole; CZM  =  ceftazidime; CSN  =  cefsulodin; CTX =  cefotaxime

### O-antigen does not Impact Patterns of Antibiotic Sensitivity in PBP Mutants

**Figure 2:**
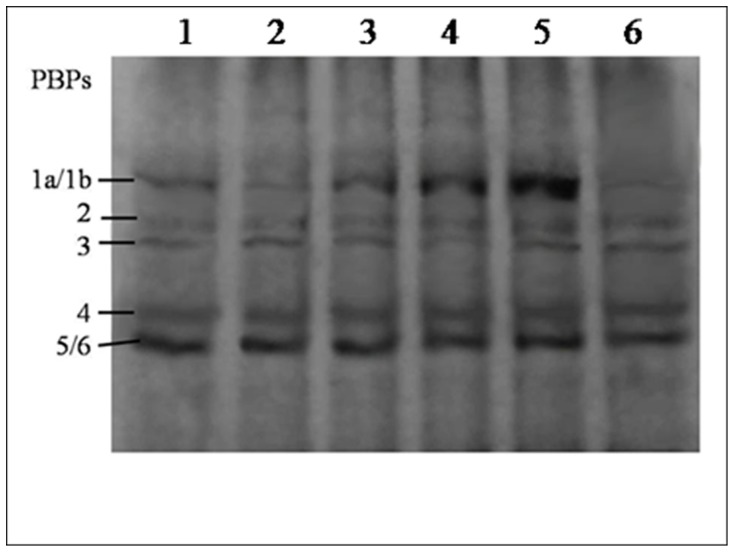
Labeling of penicillin-binding proteins with fluorescent penicillin. Total protein content (∼300 µg) were labeled with Bocillin FL (50 µM) and analyzed through 12% SDS-PAGE (100 µg/lane). Lane 1: *Escherichia coli* 2443; lane 2: 2443Δ*mrcB*; Lane 3, 4 and 5: 2443Δ*mrcB*/pSAD588-1 induced by 0.05%, 0.1% and 0.2% arabinose respectively; Lane 6: 2443Δ*mrcA*.

It is not known whether the preferred target of cefsulodin is PBP1a or PBP1b, so *mrcA* and *mrcB* genes were deleted separately from *E. coli* K12 strains that either lacked or contained O-antigen (CS109 and 2443, respectively). PBP loss was confirmed by Bocillin FL labeling (Figure 2). The effect of O-antigens on β-lactam sensitivity of the PBP mutants was tested and the patterns of alteration in β-lactam sensitivity were found identical for the strains, regardless of the presence of O-antigens (Table 3). The only difference was that the strains derived from *E. coli* 2443 were comparatively 2 to 4 times more sensitive to the penicillin group but not to the cephalosporin group of antibiotics [6]. As the pattern of sensitivity alterations was similar in both the mutants, unless otherwise specified, further experiments were carried out with the O-antigen positive PBP mutants.

**Table 3:** β-lactam^a^ sensitivities of *Escherichia coli* strains and their mutants.

MIC values (mg/L) of β-lactam antibiotics tested														
Strains	AMX	AMP	PIP	PNG	CDL	CLN	CTN	CCR	CXN	CML	CZN	CZM	CSN	CTX
CS109	8	8	2	250	32	8	16	4	4	2	4	4	32	1
CS109Δ*mrcA*	8	8	2	250	32	8	16	4	4	2	4	4	32	1
CS109Δ*mrc*B	4	4	0.125	125	2	0.5	1	0.5	0.5	0.25	0.125	0.125	1	0.06
CS109Δ*mrcB*/pSAD588-1	8	8	2	250	32	8	16	4	4	2	4	4	32	1
2443	4	2	1	125	16	8	16	4	4	2	4	4	32	1
2443Δ*mrc*B	2	1	0.06	64	1	0.5	1	0.5	0.5	0.25	0.125	0.125	1	0.06
2443Δ*mrcA*	4	2	1	125	16	8	16	4	4	2	4	4	32	1
2443Δ*mrc*B/pSAD588-1	4	2	1	125	16	8	16	4	4	2	4	4	32	1
2443Δ*mrcA*/pFS1A1	4	2	1	125	16	8	16	4	4	2	4	4	32	1

a AMX  =  amoxicillin; AMP  =  ampicillin; PIP  =  piperacillin; PNG  =  penicillin G; CDL  =  cefadroxil; CLN  =  cefalexin; CTN  =  cefalothin; CCR  =  cefachlor; CXN  =  cefoxitin; CML  =  cefamandole; CZN  =  cefoperazone; CZM  =  ceftazidime; CSN  =  cefsulodin; CTX  =  cefotaxime.

### Deletion of PBP1a is Unable to Sensitize *E. coli* Cells to β-lactams

It is believed that PBP1a and 1b compensate each other functionally for transglycosylase and transpeptidase activity; thus, in the absence of PBP1a, PBP1b can compensate its function and vice versa [11]. Therefore, to check the effect of PBP1b deletion on the sensitivity of β-lactam antibiotics, we used the CS109Δ*mrcB* and 2443Δ*mrcB* strains. These strains were sensitive to the representative antibiotics of various generations of cephalosporins, with the change in sensitivity level ranging from 16 to 32 fold, as compared to their respective parent strains (Table 3). The results indicate that in the absence of PBP1b, the intact PBP1a protein may not possess sufficient activity to compensate the physiological functions of PBP1b [15], [16]. Next, to check whether PBP1a deletion has a similar role in altering β-lactam sensitivity, the MIC values were determined for CS109Δ*mrcA* and 2443Δ*mrcA*. However, no change in β-lactam sensitivity was observed for either of the PBP1a mutants indicating that the intact PBP1b protein present in the Δ*mrcA* mutants is able to compensate functionally for the PBP1a deletion (Table 3).

**Table 4:** The MIC values in combination with Cefsulodin (4 µg ml^−1^) against *E. coli.*

*E. coli* strains		AMX	AMP	PIP	PNG	CDL	CLN	CTN	CXN	CCR	CFL	CZN	CZM	CTX
2443	A	4	2	1	125	16	8	16	4	4	2	4	4	1
	B	1	0.5	0.125	64	4	2	4	1	1	0.5	0.5	0.5	0.125
CS109	A	8	8	2	250	32	8	16	4	4	2	4	4	1
	B	4	4	0.25	125	8	2	4	1	1	0.5	0.5	0.5	0.125

AMX  = Amoxicillin; AMP  = Ampiocillin; PIP  = Piperacillin; PNG  = Penicillin G; CDL  = Cefadroxil; CLN  = Cefalexin; CTN  = Cefalothin; CXN  = Cefoxitin; CCR  = Cefaclor; CFL  = Cefamandole; CZN  = Cefoperazone; CZM  = Ceftazidime and CTX  = Cefotaxime.

A: The sensitivities in absence of Cefsulodin; B: The sensitivities in presence of Cefsulodin (4 µg ml^−1^).

**Figure 3:**
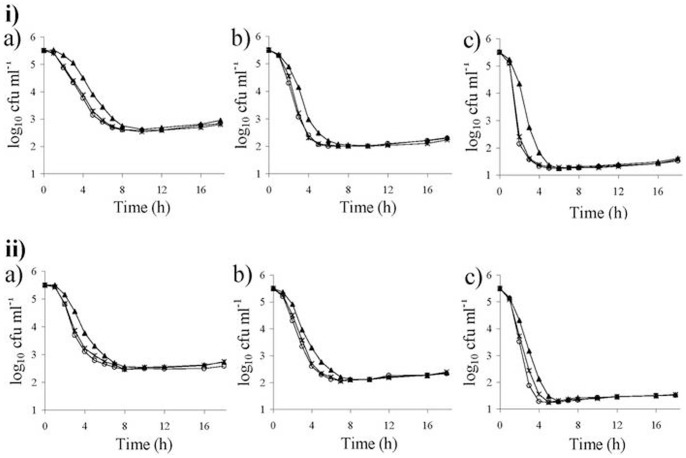
Graphical representation of Time–kill analyses. ‘▴’ *E. coli* 2443, ‘o’ 2443Δ*mrcB*, ‘x’ 2443 plus cefsulodin (4 mg/L) in presence (a) 1×MIC, (b) 2×MIC, (c) 4×MIC of (i) ampicillin and (ii) cefadroxil.

### Expression of PBP1b in Trans Restores the Lost β-lactam Resistance

From our results, we inferred a possible involvement of PBP1b in altering β-lactam sensitivity. To strengthen this hypothesis, we checked whether the expression of PBP1b in trans could reverse the augmented β-lactam sensitivity in CS109Δ*mrcB* and 2443Δ*mrcB* (Table 3). Expression of *mrcB* gene from plasmid pSAD588-1 (Table 1) reversed the lost β-lactam sensitivity in both the strains. However, no change in β-lactam sensitivity was observed upon expressing *mrcA* (from plasmid pFS1A1) in 2443Δ*mrcA*. Therefore, the results obtained from both the deletion and complementation experiments demonstrate that PBP1b is involved in maintaining an intrinsic β-lactam resistance, especially to cephalosporins.

### Cefsulodin Binding Efficacy of PBP1a is Ten Times Higher than that of PBP1b

To understand the biochemical origins of the variation in the physiological functions of PBP1a and PBP1b, their binding efficacy for cefsulodin was determined through a competition assay between cefsulodin and Bocillin FL using the strains 2443Δ*mrcA* and 2443Δ*mrcB*. The relative efficacy of binding of cefsulodin to PBP1s was determined by their ability to inhibit the binding of Bocillin FL by 50%, also known as IC_50_ value (50% inhibitory concentration). Cefsulodin was found to interact specifically with PBP 1a and 1b. The band intensity of 2443Δ*mrcB* (where PBP1a was intact) showed 50% inhibition in presence of 10 µM cefsulodin, while 2443Δ*mrcA* (where PBP1b was intact) showed 50% inhibition in presence of 100 µM cefsulodin. Assessment of the IC_50_ of cefsulodin for the PBP1s revealed that PBP1a has an IC_50_ value that is approximately 10 times lower than that of PBP1b. In other words, PBP1a is 10 times more sensitive to cefsulodin than PBP1b, which resembles the result reported by Ramachandran et al [17].

### Sub-inhibitory Levels of Cefsulodin in Combination with β-lactams Mimics PBP1b Deletion

The efficiency of sub-inhibitory concentrations of cefsulodin in combination with other β-lactams was tested. As described above, 4 mg/L of cefsulodin was most effective in sensitizing the clinical isolates to β-lactams. The MIC values obtained for each antibiotic tested in combination with cefsulodin (4 mg/L), resembled the pattern observed with *mrcB* deletion. However, the enhancements in sensitivity were in a range from 2 to 8 fold for the entire set of antibiotics tested (Table 4).

To check the effect of 4 mg/L cefsulodin in combination with other β-lactams, we evaluated the killing rate of parent and PBP1b mutant strains using 1× and 2× MICs of ampicillin and cefotaxime as representatives of penicillin and cephalosporin groups, respectively. The results were plotted as log cfu/mL versus time [14] (Figure 3). Interestingly, the killing rate of 2443Δ*mrcB* in the absence of cefsulodin was similar to that of the 2443 parent strain in presence of 4 mg/L cefsulodin. Therefore, β-lactam in combination with 4 mg/L cefsulodin showed an effect similar to PBP1b loss in *E. coli*.

Overall, based on the obtained results, it can be speculated that in a cell where both the PBPs are intact, cefsulodin inhibits PBP1a at a concentration 10 times lower than PBP1b. If concentration of cefsulodin is sub-optimal, majority of PBP1b remain viable for its enzymatic functions. Similar situation prevails for PBP1a deletion mutants leading to unaltered MIC values. However, if the sub-inhibitory dose of cefsulodin is sufficient to inhibit PBP1b (at least partially), the availability of functional PBP1b diminishes. In this situation, the cells become more vulnerable to β-lactams that target other essential PBPs. Therefore, a sub-inhibitory concentration of cefsulodin would be sufficient to inhibit PBP1a and at least partially inhibit PBP1b. On the other hand, when PBP1b is deleted, the intact PBP1a would be inhibited by a much lower dose of cefsulodin as compared to PBP1b, which explains the reason for enhanced β-lactam sensitivity of PBP1b deletion mutant.

### Conclusion

It is inferred that PBP1b is involved in altering β-lactam sensitivity, especially for antibiotics of the cephalosporin group. We suggest that by deleting *mrcB* or its homologs, and/or by applying a sub-inhibitory level of cefsulodin (4 mg/L), the bacterial cells, regardless of the presence of β-lactamases, can be sensitized against conventional β-lactam agents. Further studies in this area may expand our knowledge of combinatorial therapy using cefsulodin as a key component.
